# Using a modified double deep image prior for crosstalk mitigation in multislice ptychography

**DOI:** 10.1107/S1600577521003507

**Published:** 2021-05-19

**Authors:** Ming Du, Xiaojing Huang, Chris Jacobsen

**Affiliations:** a Advanced Photon Source, Argonne National Laboratory, Argonne, IL 60439, USA; b National Synchrotron Light Source II, Brookhaven National Laboratory, Upton, NY 11973, USA; c Department of Physics and Astronomy, Northwestern University, Evanston, IL 60208, USA; d Chemistry of Life Processes Institute, Northwestern University, Evanston, IL 60208, USA

**Keywords:** ptychography, multislice, neural network, image processing, artifact removal

## Abstract

Multislice ptychography is a high-resolution microscopy technique used to image multiple separate axial planes using a single illumination direction. However, multislice ptychography reconstructions are often degraded by crosstalk, where some features on one plane erroneously contribute to the reconstructed image of another plane. Here, the use of a modified ‘double deep image prior’ (DDIP) architecture is demonstrated in mitigating crosstalk artifacts in multislice ptychography. Utilizing the tendency of generative neural networks to produce natural images, a modified DDIP method yielded good results on experimental data. For one of the datasets, it is shown that using DDIP could remove the need of using additional experimental data, such as from X-ray fluorescence, to suppress the crosstalk. This method may help X-ray multislice ptychography work for more general experimental scenarios.

## Abstract 2

Using the ‘deep image prior’ characteristic of generative neural networks, a post-processing method that mitigates the inter-slice crosstalk issue in multislice ptychography has been developed. Under certain scenarios, crosstalk can be suppressed effectively in slice images reconstructed using ptychographic diffraction data alone.

## 1. Introduction   

In ptychography, a spatially limited coherent probe is scanned across multiple transverse positions; the far-field diffraction patterns are then used to reconstruct the complex optical transmittance of a planar object (Faulkner & Rodenburg, 2004 ▸). Multislice ptychography (Maiden *et al.*, 2012 ▸; Tsai *et al.*, 2016 ▸) is an extension of this approach for imaging multiple axial planes each separated by a distance *z*_DoF_ greater than the depth of field (DoF) of (Born *et al.*, 1999 ▸; Gilles *et al.*, 2018 ▸) 

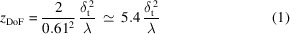

where δ_t_ is the transverse spatial resolution. In multislice ptychography, the probe illumination function at each probe position is modulated by the first axial plane, after which Fresnel propagation is used to bring it to the next plane, and so on until the far-field diffraction intensity is obtained.

When the contrast of upstream planes is significant enough that the first Born approximation is violated, the illumination of downstream planes is significantly affected; if incorrectly accounted for in a reconstruction algorithm, this can lead to crosstalk between the images from these separate planes. Even with low contrast objects, if the axial separation between object planes is only a small multiple of *z*_DoF_, Fresnel propagation alone may be insufficient to cleanly reconstruct the two planes correctly. This can be seen in a 12 keV X-ray multislice ptychography experiment where crosstalk was observed in δ_t_ = 9.2 nm images of objects on two planes separated by 10 µm, or 2.3 × *z*_DoF_ = 4.4 µm in this case (Öztürk *et al.*, 2018 ▸).

Given that hard X-ray microscopy is well suited to imaging objects in this thickness range (Du & Jacobsen, 2018 ▸), this limitation of multislice ptychography becomes important to overcome. Alternative approaches include ptychographic tomography for objects that do not extend in depth beyond *z*_DoF_ at any rotation angle (Dierolf *et al.*, 2010 ▸), or multislice ptychographic tomography of thicker objects where propagation is used to compensate for Fresnel diffraction blurring but images of separate planes are not required (Van den Broek & Koch, 2012 ▸; Kamilov *et al.*, 2015 ▸; Li & Maiden, 2018 ▸; Gilles *et al.*, 2018 ▸; Du *et al.*, 2020*a* ▸); however, both of these approaches require images obtained over multiple object rotation angles. The more extensive data collection required for these tomographic approaches is not always feasible or desirable, so it remains important to overcome crosstalk effects in single-viewing-direction multislice ptychography of separate object planes.

Many ptychographic beamlines at synchrotron light sources are equipped with both an area detector for recording far-field coherent diffraction data and an energy-dispersive detector for recording X-ray fluorescence (XRF) signals in the same scan of the illumination probe. Unlike ptychography, fluorescence imaging is an incoherent process with a spatial resolution limited by the focusing optic used; however, XRF can provide low spatial frequency information of a sample with distinct distributions of chemical elements. This approach has been used to provide low-crosstalk reconstructions of an upstream plane object consisting of an Au zone plate structure and a downstream plane consisting of NiO particles mounted on a silicon nitride window (Huang *et al.*, 2019 ▸). In this case, the Ni XRF image was used to generate an initial guess of the object on the downstream plane, as well as to subtract the spectrum of the NiO object’s ‘ghost image’ from an initial reconstruction of the upstream plane, after which a multislice ptychographic reconstruction was allowed to proceed. The resulting images [shown in Figs. 3(*a*) and 3(*b*) of Huang *et al.* (2019 ▸)] indeed show almost no crosstalk between the reconstructed images at the two axial planes.

While the XRF-aided reconstruction has been shown to be effective, its limitation is also apparent: if the chemical composition of objects on the different axial planes is similar, then XRF can no longer provide strict object separation. Therefore, it is valuable to explore alternative methods to suppress crosstalk without using XRF data. In fact, the crosstalk separation problem resembles the well known problem of blind source separation (BSS) in signal processing (Cao & Liu, 1996 ▸). In the BSS problem, one begins with *N* measurements **y** = [*y*_1_(*t*), *y*_2_(*t*),…, *y*_*N*_(*t*)], where each measurement is a linear superimposition of *M* source signals **s** = [*s*_1_(*t*), *s*_2_(*t*),…, *s*_*M*_(*t*)] with a unique set of weighting factors *w*_*n*,*m*_ so that one obtains measured data of *y*_*n*_(*t*) = 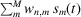 . The goal in this case is to solve the linear system 

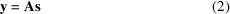

so as to obtain the source signals **s**. The problem can be overdetermined, underdetermined, or exactly determined depending on the relative values of *M* and *N*. Separating out all *M* sources requires *N* ≥ *M*. Obviously, for multislice ptychography, *N* = *M*, which is a necessary condition for all ‘clean’ slices to be solved from phase retrieved slices containing crosstalk.

The complication for multislice ptychography is that the ghost features are not a simple superimposition added onto an affected slice but rather a filtered version of the real features after losing information in certain spatial frequency bands. For example, the ghost particles in one axial plane of Fig. 4(*a*) of Öztürk *et al.* (2018 ▸) appear like a low-pass filtered version of features in the other axial plane. This band loss has to be taken into account before separating the ghost features. Moreover, for a BSS problem to be solved successfully, the rows of **A** in equation (2) should be linearly independent. In the case of multislice ptychography, that requires sufficient differentiation between real and ghost features in the axial slices. When the slice spacing is large, this condition is usually easy to satisfy. However, if the slice separation is too small, the weak probe variation between adjacent slices makes them dificult to be cleanly reconstructed when starting from a random guess, since this can yield retrieved slices that are too similar to each other. Under this scenario, we may relax our constraint and allow the use of XRF data to assist with the initial phase retrieval. However, it turns out that even with good initial guesses aided by XRF, one is still unable to fully eliminate inter-slice crosstalk without a very careful search of reconstruction parameters and reconstruction algorithms. For example, to obtain Figs. 3(*a*) and 3(*b*) of Huang *et al.* (2019 ▸), many efforts were made to optimize the algorithm and parameters. Before doing that, a standard reconstruction yielded images with considerable crosstalk as shown in Fig. 4(*a*) of our paper. We demonstrate here that crosstalk can be greatly reduced, so that in both situations (large separation without using XRF, and small separation with XRF) the crosstalk can be mitigated using a neural network algorithm based on a ‘double deep image prior’, or ‘double-DIP’ (DDIP).

In the deep image prior (DIP) approach (Ulyanov *et al.*, 2018 ▸), images in the forward model are generated from a generative neural network, so that the network itself functions to provide prior knowledge to the system. This is because a deep neural network prefers generating ‘natural images’ with lower patch-wise entropy, rather than those with higher patch-wise entropy (Gandelsman *et al.*, 2018 ▸). In Ulyanov *et al.* (2018 ▸), DIP has been demonstrated to perform well for a series of tasks such as denoising and deblurring. As described in the cited paper, DIP is a type of ‘untrained’ neural network, which means that, instead of training the networks on a large dataset and then using the trained networks for non-iterative prediction, one ‘trains’ (or ‘fits’, which might be a more proper term) the neural network to solve one particular problem using only the data pertaining to that problem and an explicit model describing the image degradation process, without any labels or ground truths. There is no separated prediction phase in addition to the iterative training phase, as the fitting already results in a network that can generate the restored image (and it *only* learns to generate that image). When one applies the neural network architecture to another problem, it needs to be ‘fitted’ again using the data of that problem. It might be helpful to analogize the workflow of the untrained neural network approach to that of conventional model-based inverse problem solvers, such as ePIE (Maiden & Rodenburg, 2009 ▸). These model-based solvers also perform iterative optimization for each different problem, without training on a large dataset in advance. However, with DIP, instead of directly solving for the restored images, we solve for the parameters in the generative networks which in turn generate the restored images, so that we can exploit the prior knowledge coming with these neural networks. In other words, the untrained DIP approach is more properly classified as a model-driven method, instead of a data-driven method like traditional neural network-based approaches.

Since the ‘untrained’ usage of DIP requires iterative fitting for each distinct problem, one may question its efficiency. The untrained DIP approach does not enjoy the non-iterative prediction as in data-driven neural networks. However, since for each problem the DIP networks are fitted only on the data pertaining to a single problem, the iterative fitting time is generally short, and a moderate-scale network that can fit into the memory of a modern GPU would usually suffice. Also, the generalizabilty of a DIP-based algorithm is not constrained by the training set; that is, we are not concerned with overfitting. This initial demonstration of DIP (Ulyanov *et al.*, 2018 ▸) used an encoder–decoder structure which learns to map an input tensor to an image with the same spatial dimension. By using an encoder–decoder network with skip connections linking the encoder part and the decoder part, one can lead the network to generate images with structure at multiple spatial scales, thus better capturing the characteristics of natural images.

Building upon this initial work, it has been shown that the use of multiple DIP networks can achieve improved outcomes on a series of layer decomposition problems including image dehazing, image segmentation, and transparency separation whose goal is to separate out multiple individual natural images from blends of them (Gandelsman *et al.*, 2018 ▸). All these tasks can be carried out using a similar architecture: if there are two layers to be separated, one can use two DIP networks to generate two distinct images, and use a third DIP to generate either a mask or a constant blending ratio. The architecture is thus named ‘double-DIP’ (DDIP) after the two image-generating DIPs. Using the generated images and the mask or ratio, one can synthesize a blended image, and train the networks to minimize a loss function measuring the mismatch between the synthesized image and the original blended images. The preference of DIPs to generate natural images means that the local patches consisting of the images they generate usually have lower empirical entropy, which is an indication that these images are more likely to be unblended ‘single’ images. Additionally, prior work (Gandelsman *et al.*, 2018 ▸) also included an exclusion term in the loss function, which penalizes the correlation between the spatial gradients of the generated images. This further suppresses the crosstalk in the output images.

Therefore, one can expect that the DDIP architecture can function effectively in the multislice ptychography crosstalk separation problem. In view of the additional band loss complication of the ghost features, we modified the DDIP architecture from the original design of Gandelsman *et al.* (2018 ▸). The network architecture will be introduced in more detail in Section 2. In Section 3, we will show the results obtained using DDIP on two datasets, each representing one of the slice spacing situations mentioned above.

## 2. Methods   

### 2.1. Algorithm   

**Figure 1:**
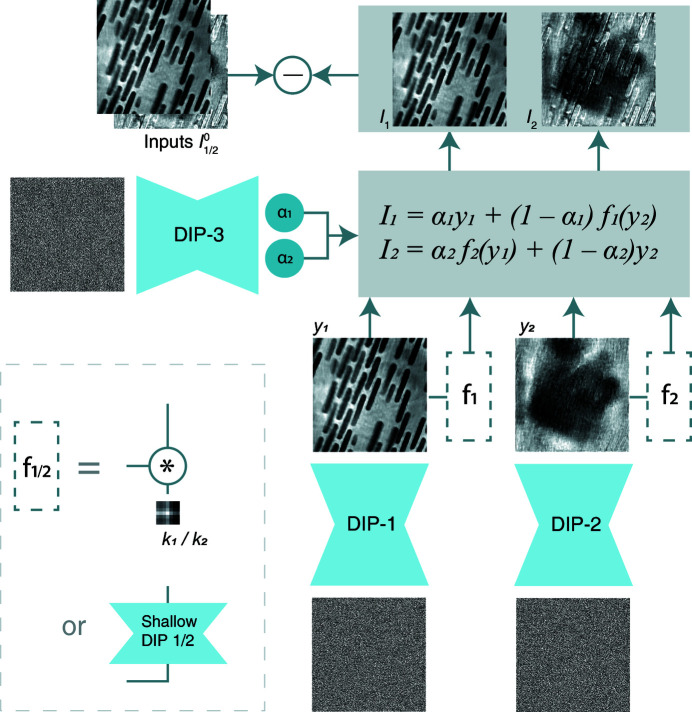
The ‘double DIP’ model used in this work. Building on prior work (Gandelsman *et al.*, 2018 ▸), the outputs of both image-generating deep image priors (DIPs; as shown in Fig. 2 ▸) are filtered by function *f*_1/2_ to account for the partial band transfer of superimposed images. *f*_1/2_ can be either a single-layer filter, or a shallow three-level DIP network.

**Figure 2:**
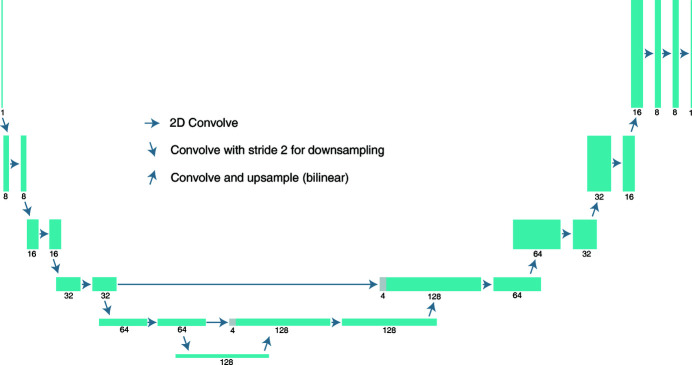
Architecture of a DIP network used in the DDIP model. Numbers underneath tensor blocks indicate the number of channels.

The overall model structure of our modified DDIP is shown in Fig. 1 ▸. The two image-generating DIPs, labeled DIP-1 and DIP-2, are of the same ‘U-Net’-like architecture (Ronneberger *et al.*, 2015 ▸), as shown in Fig. 2 ▸. The kernel size used in all 2D convolutional layers is 5 × 5; an exception is the skip connections, where 1 × 1 kernels are used. The input/output numbers of channels of these convolutional layers are shown in the figure. A leaky ReLU is used after each 2D convolutional layer as the activation function. The inputs to both DIPs, *z*_1_ and *z*_2_, are mono-channel tensors of random numbers that are uniformly sampled between −0.5 and 0.5 and have the same height and width as the original images. The DIP that generates the constant weighting factor, DIP-3, adopts the same architecture as DIP-1 and DIP-2 except that the input and output numbers of channels are two. During training, DIP-1 and DIP-2 learn to map *z*_1_ and *z*_2_ to *y*_1_ and *y*_2_ which are supposed to be the ‘clean’ slice images. For DIP-3, the values of the central pixels from both output channels are used as the blending weights α_1_ and α_2_. In Gandelsman *et al.* (2018 ▸), a linear combination is used to synthesize the blended images *I*_1_ and *I*_2_ from generated images *y*_1_ and *y*_2_, *i.e.* *I*_1_ = α_1_ *y*_1_ + (1 − α_1_)*y*_2_, and *I*_2_ = α_2_ *y*_1_ + (1 − α_2_)*y*_2_. In our case, to account for the band loss of the ghost features, we pass the images of the source of crosstalk through an additional function *f*_1_ or *f*_2_, giving 

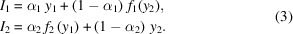

**Figure 3:**
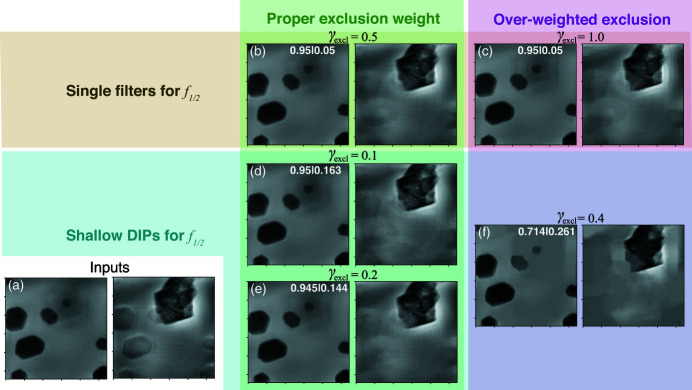
Input images (*a*) and separation results (*b*)–(*f*) of the Au/NiO dataset. The results were obtained with *f*_1/2_ set to either single filters or shallow DIPs. For each case, several γ_excl_ values were tested. Ghost features are effectively suppressed with a proper setting for γ_excl_. However, when γ_excl_ is too large, fine details of the features are smeared out. The final values of α_1_|α_2_ are indicated at the upper right corners of the corresponding subplots. The values of α hold steady except when we use shallow DIPs for *f*_1/2_ and over-weight the exclusion loss, in which case the ratio α_1_/α_2_ decreases significantly.

We explored two types of choices for *f*_1_ and *f*_2_. Before discussing the choices for these functions, one can see in Figs. 3 ▸(*a*) and 4 ▸(*a*) that the ghost images from more strongly scattering materials (*e.g.* gold) appear like the high-pass filtered version of the real features. On the other hand, the more weakly scattering materials (*e.g.* NiO) contribute to the crosstalk with a low-passed version of the real features. Thus, one can define *f*_1_ and *f*_2_ as two single-kernel filtering functions, which can be implemented through 2D convolution,

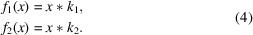

Based on the appearance of the original images, *k*_1_ and *k*_2_ can be initialized to be a low-pass or high-pass kernel. During training, their values are optimized along with the DIP parameters. For our results to be shown in Section 3 where both cases are consisted of one slice with low-pass crosstalk and another with high-pass crosstalk, we set *k*_1_ to be a 7 × 7 uniform filter, and *k*_2_ to be a 7 × 7 kernel containing a five-point Laplacian filter.

A single filtering kernel may not be able to capture the band loss at various spatial scales. Therefore, a second way is to set *f*_1_ and *f*_2_ as another two shallow DIPs with downsampling and skip connections. In our implementation, we used a three-level DIP with the same kernel size as DIP-1, -2, and -3, so that there are three downsampling/upsampling operations, each with a factor of two. However, the number of channels of intermediate tensors is always one. Additionally, skip connections are used at all three spatial scales in order to prevent the loss of high-frequency information. These shallow DIPs are initialized using uniform random numbers, and the parameters are optimized along with the ‘major’ DIPs during training.

With these, we can now formulate the loss function which contains a data mismatch term measuring the difference between the synthesized images *I*_1/2_ and the original images 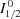 . Additionally, as indicated by Gandelsman *et al.* (2018 ▸), it is also essential to employ an exclusion loss which penalizes the correlation of the spatial gradients of *y*_1_ and *y*_2_ at multiple spatial scales. The values of α_1_ and α_2_ are also penalized for drifting away from 0.5 at the first 100 epochs of the algorithm in order to stabilize their values against the random input and network initialization. Thus, the full loss function (for a two-slice separation task) is written as 

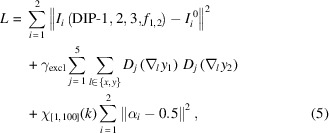

where γ_excl_ is a constant weight of the exclusion loss term, *D*_*j*_ is the downsampling function that downsamples the image in its argument by a factor of 2^*j*−1^, ∇_*l*_ *y* denotes the spatial gradient of *y* along direction *l* (either *x* or *y*), and χ_[1,100]_(*k*) is a step function of epoch number *k* that returns 1 when *k* ≤ 100, and 0 otherwise.

Our model is trained on an HP Z8 G4 workstation with two Intel Xeon Silver 4108 CPUs and two NVIDIA Quadro P4000 GPUs, although the model is run using only one GPU each time. *PyTorch* (Paszke *et al.*, 2019 ▸) is used for automatic differentiation. The code is available on https://github.com/mdw771/ddip4ptycho.

### 2.2. Beamline experiments   

The datasets used in both cases shown in Section 3 were acquired at the Hard X-ray Nanoprobe beamline (3-ID) of the National Synchrotron Light Source II at Brookhaven National Laboratory.

The first dataset involves a synthetic sample, where Au nanoparticles and NiO particles are deposited on both sides of a 10 µm-thick Si wafer. We will hereafter refer to this dataset as the Au/NiO dataset. The dataset was collected with a beam energy of 12 keV and a transverse resolution δ_t_ = 7.3 nm, which, according to equation (1), gives *z*_DoF_ = 2.8 µm. The 10 µm slice spacing is therefore about 3.6 times larger than *z*_DoF_. The multislice reconstruction result of this dataset was published earlier by Öztürk *et al.* (2018 ▸), which can be referred to for more experimental details. Similar to Öztürk *et al.* (2018 ▸), we assume two slices in the sample, which, respectively, correspond to the Au layer and the NiO layer.

The second dataset, described here as the ZP/NiO dataset, also involves a two-slice sample that has been previously published (Huang *et al.*, 2019 ▸). In this case, Au zone plate structures and NiO particles are deposited on both sides of a 500 nm-thick silicon nitride membrane. The beam energy and transverse resolution on the first slice are 12 keV and 8.7 nm, giving *z*_DoF_ = 3.9 µm. Hence, the slice spacing is just about 0.13 of the DoF.

## 3. Results   

### 3.1. Large-spacing separation for Au/NiO data   

The crosstalk-contaminated slice images of the Au/NiO dataset were reconstructed using an adaptive momentum based algorithm in a tool we developed called *Adorym* (Du *et al.*, 2020*b* ▸). The phase retrieval was initialized using Gaussian randoms, without using the XRF data. Because the slice spacing is 3.6 times larger than *z*_DoF_, our multislice reconstruction algorithm is able to provide reconstructions of both slices with the ‘true’ features of each slice resolved sharply, but they also exhibit obvious ghost features due to the crosstalk. Next, we cropped a 272 × 272 pixel area that has full probe overlap from each slice [Fig. 3 ▸(*a*)], and passed the slices to DDIP as 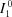 and 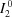 .

We performed five test runs with *f*_1/2_ set to use either shallow DIPs or single filters for *f*_1/2_, and with different values of γ_excl_. Each parameter combination was run for 10000 epochs. When using shallow DIPs for *f*_1/2_, the peak GPU memory usage was 439 MB, and each run took around 30 min to complete. The results are shown in Figs. 3 ▸(*b*)–3(*f*), where the final values of α_1_ and α_2_ are indicated at the top right corners of the corresponding subplots as α_1_|α_2_. The dynamic range of all plots is set to [μ − 4σ,  μ + 4σ], with μ and σ being the image mean and standard deviation.

Since the ghost image on slice 1 of the NiO particle (which is in fact on slice 2) is very blurry, it appears like a subtle change in the image background. Under all tested parameter settings, DDIP barely affected the presence of this faint region. This can be explained by the nature of deep image priors: as noted by Gandelsman *et al.* (2018 ▸), generative neural networks tend to generate images that have a smaller empirical entropy across its local patches; in other words, the generated images tend to have ‘strong internal self-similarity’. Since the ghost feature on slice 1 is very smooth, it is hard for DIPs to exclude it from the generated image. However, the ghost features on slice 2 are sharp and have a much higher variance. They make the local patches of the image more complicated and more ‘unlike’ each other, so DIP tends to generate images that are free of these artifacts. Therefore, the improvement of slice 2 is obvious. The effect on slice 2 is also largely dependent on γ_excl_ regardless of whether *f*_1/2_ is set to use shallow DIPs or single filters. When using single filters for *f*_1/2_, the setting of γ_excl_ = 0.5 can provide an apparent mitigation of the crosstalk coming from slice 1, where the sharpness and contrast of the ghost Au particles are greatly reduced. Increasing γ_excl_ to 1.0 suppresses the ghost features even further, but it also starts to destroy details in the ‘true image’ of the NiO particle. In particular, the regions in the NiO particle that overlap with ghost Au particles are severely smeared. Given such high values of γ_excl_, the correlation of gradients is over-penalized and the algorithm tends to reduce the spatial gradient of slice 2 at the overlapping regions to 0, resulting in flattened areas.

Improved results are obtained when we switch *f*_1/2_ to use shallow DIPs. In Fig. 3 ▸(*d*), when γ_excl_ = 0.1, the crosstalk suppression on slice 2 is nearly as effective as for Fig. 3 ▸(*b*) with single filters and γ_excl_ = 0.5. Increasing γ_excl_ to 0.2 slightly enhances the suppression effect, surpassing the efficacy of Fig. 3 ▸(*c*) with single filters and γ_excl_ = 1.0. Moreover, comparing Figs. 3 ▸(*c*) and 3 ▸(*e*) reveals that using shallow DIPs leads to much better preserved high-frequency details in the NiO particle. This is an expected improvement, as the multi-scale filtering with skip connections in the shallow DIPs better describes the band loss of ghost features than single filters. If one increases γ_excl_ further to 0.4, however, the images would start to lose high-frequency details as well.

The final values of blending weights for all cases are composed of a large α_1_ and a small α_2_. Based on equation (3), this indicates that *y*_1_ contributes much more than *f*_1_(*y*_2_) does to *I*_1_, while *y*_2_ contributes more than *f*_2_(*y*_1_) to *I*_2_. This is a reasonable trend as one would expect a smaller contribution from the ghost features than real features in a ‘blended’ slice. However, we should not interpret the α values as the absolute intensities of the ghost or real features present in *I*_1_ or *I*_2_, since the mean intensities of *y*_1_, *y*_2_, *f*_1_(*y*_2_), and *f*_2_(*y*_1_) can vary as well. On the other hand, the α pair may be used as an indication of the fidelity of the result. In Fig. 3 ▸(*f*), where the details of the features are obviously undermined, the final value of α_1_ is much lower than other results with better preserved features, while α_2_ is much higher. Since the algorithm always tries to minimize the mismatch between *I*_1/2_ and 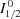 where the latter is fixed, unusual α values point to unusual value ranges of the outputs of DIP-1/2 and *f*_1/2_, implying that the generated images might be highly aberrated.

### 3.2. Small-spacing separation for ZP/NiO data   

**Figure 4:**
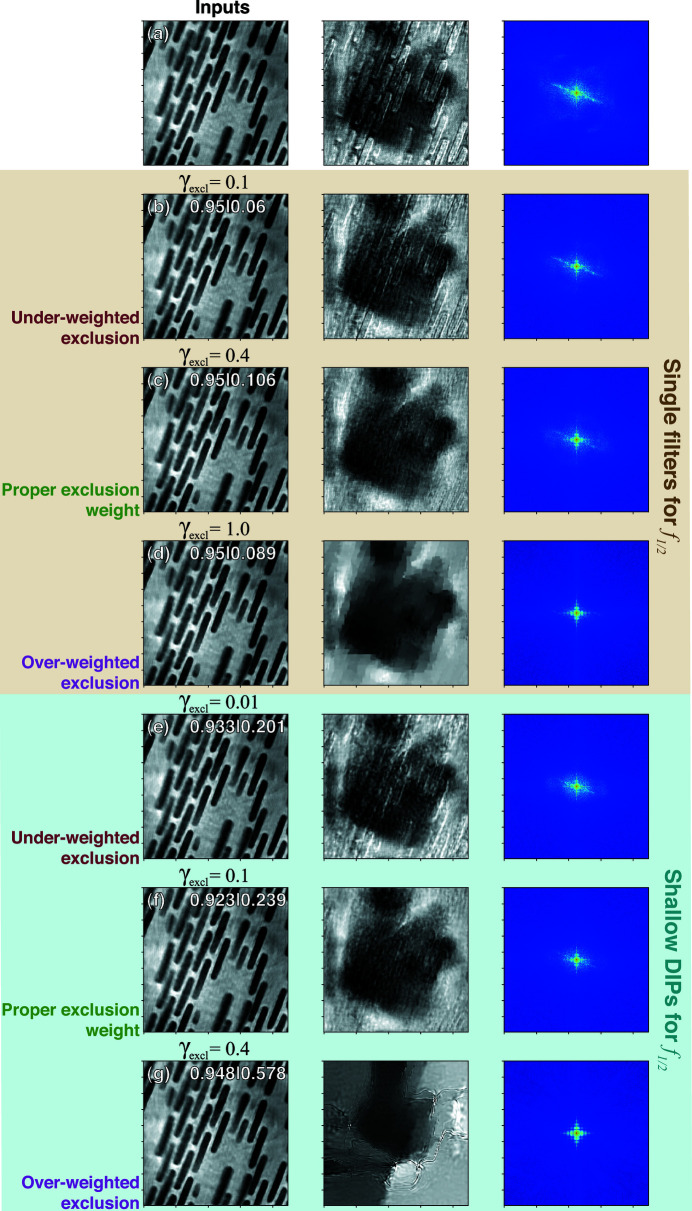
Input images (*a*) and separation results (*b*–*g*) of the ZP/NiO dataset. Like in the cases shown in Fig. 3 ▸, the results were obtained with *f*_1/2_ set to either single filters or shallow DIPs. The final values of α_1_|α_2_ are indicated at the upper right corners of the corresponding subplots. While the influence of γ_excl_ on slice 1 is minimal, it greatly affects the balance between separation effectiveness and image resolution for slice 2. The rightmost column shows the normalized power spectra of slice 2, plotted as 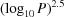 (where *P* is the original power spectrum). A slanted streak corresponding to the periodicity of the zone plate’s ghost features can be seen obviously in the input image’s spectrum. In the outputs of the DDIP (*i.e.* *y*_2_), the spectrum density of this streak becomes much lower. Also, we again see that, when we use shallow DIPs for *f*_1/2_ and over-weigh the exclusion loss, a smaller α_1_/α_2_ ratio is yielded.

The 500 nm slice spacing in the ZP/NiO dataset is only about 0.13 times the DoF. As such, our attempt of reconstructing both slices using random initial guesses yielded two slices that are largely undifferentiated. The superimposed features on both slices are mixed with an almost identical ratio, and the band loss of ghost features is very small. Images like this could hardly provide enough diversity of measurement in order to solve the BSS problem. Therefore, it becomes essential to employ the XRF data as additional prior knowledge to the reconstruction algorithm. As mentioned earlier, the slice images to be separated were obtained using the XRF-aided method described by Huang *et al.* (2019 ▸), where the XRF map of Ni is used to reduce the contrast of NiO in the single-slice reconstruction, leaving the Au zone plate structure, and the NiO-removed Au image and the re-sampled Ni XRF map are used as the initial guess for the first and second slice, respectively, for the subsequent multislice ptychographic phase retrieval. Without dedicated parameter tuning and algorithm search, standard phase retrieval could not provide well separated slices; instead, it yielded the slice images shown in Fig. 4 ▸(*a*), where slice 2 is heavily affected by the ghost images from the Au zone plate structures on slice 1. Our goal is to show that, even though XRF data have to be used, DDIP can provide better separated images based on this result, so that the excessive amount of phase retrieval parameter tuning may be avoided.

We again tested several γ_excl_ values with *f*_1/2_ using shallow DIPs or single filters. 10000 epochs are run for each case. The input image size is 448 × 448. When using shallow DIPs, the peak memory usage is 1130 MB, and it took 37 min to complete the training. On the other hand, when using single filters, the total walltime becomes 31 min, though the peak memory usage did not change significantly since the parameter size of the shallow DIPs is rather small compared with the major DIPs. The results are shown in Figs. 4 ▸(*b*)–4(*h*). Similar to what was observed with the Au/Ni dataset, the crosstalk does not significantly affect slice 1, but results in obvious ghost images on slice 2 due to the strong scattering of Au. For single filters, γ_excl_ = 0.4 [Fig. 4 ▸(*c*)] gives the best balance between crosstalk suppression and feature fidelity. Using a lower γ_excl_ of 0.1 leaves a lot of residual ghost image features, while a higher value of 1.0 results in a blocky appearance of the recovered slice 2. When using shallow DIPs, the optimal γ_excl_ is found around 0.1. If γ_excl_ is set too high, the fidelity of *y*_2_ is dramatically lost, which is accompanied by a much larger α_2_.

Since the Au zone plate structures are well aligned in the same direction, we can analyze the power spectra of the outcome *y*_2_ to evaluate the effectiveness of crosstalk suppression. These power spectra are normalized by the integrated energy, and plotted as 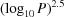 (where *P* is the original normalized power spectrum) to improve contrast. In the power spectra of the original image shown in Fig. 4 ▸(*a*), one can clearly observe a slanted streak that represents the periodicity of the zone plate ghost features. For γ_excl_ = 0.4 when using single filters, the streak is suppressed; for γ_excl_ = 0.1 when using shallow DIPs, the streak is reduced even more. Further increasing γ_excl_ in both cases cause energy to concentrate in the low-frequency region, associated with the smeared appearance of Figs. 4 ▸(*d*) and 4(*g*).

**Figure 5:**
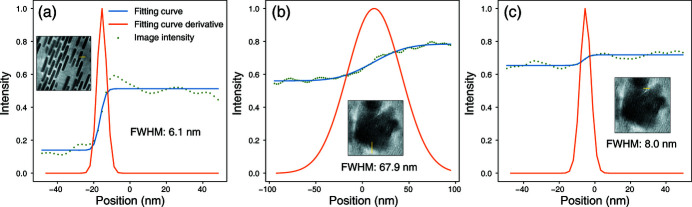
The error function fitting for the line profiles in our output images: (*a*) slice 1, and (*b*, *c*) two locations in slice 2. The location of the line profiles are indicated by the yellow lines. From the full width at half-maximum (FWHM) of the spatial derivative of the fitted error function, one can estimate the spatial resolution of these images.

Taking the results obtained using shallow DIPs for *f*_1/2_ and γ_excl_ = 0.1, we estimated the resolution of the slice images using the same method as in the earlier publication (Huang *et al.*, 2019 ▸), and made a comparison. As shown in Fig. 5 ▸(*a*), the resolution found from the full width at half-maximum (FWHM) of the spatial derivative of the fitted error function along the indicated line profile in slice 1 is 6.1 nm, better than the reported 8.7 nm of Huang *et al.* (2019 ▸). On the other hand, the FWHM for the same line position in slice 2, as shown in Fig. 5 ▸(*b*), is 67.9 nm, worse than the 15 nm reported by Huang *et al.* (2019 ▸). However, this location is not representative of the overall image. The FWHM at another location in slice 2, shown in Fig. 5 ▸(*c*), is about 8.0 nm, again revealing a good spatial resolution. Meanwhile, we should keep in mind that our method is a post-processing approach that is agnostic to the original ptychographic diffraction patterns, and the above resolution is achieved without accessing the high-frequency information in the raw diffraction data. Additionally, we note again that the reconstruction results of Huang *et al.* (2019 ▸) were obtained with careful tuning to the ptychographic reconstruction parameters. The parameter space to be tuned, and the time efforts required to achieve good results directly from ptychographic phase retrieval, can often be higher than tuning DDIP in order to yield our results. Moreover, the results of DDIP may also be used as the initial guess for a second pass of multislice ptychographic phase retrieval. With a much closer initial guess, less parameter tuning is required.

## 4. Discussion   

**Figure 6:**
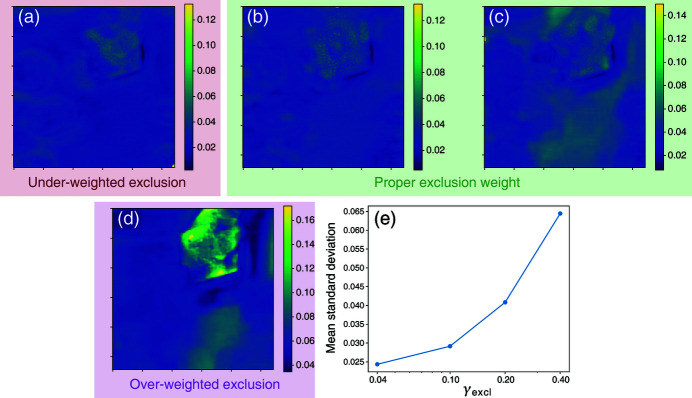
(*a*)–(*d*) Standard deviation maps of output slice 2 (*y*_2_) of the Au/Ni dataset, calculated from 20 independent runs, for γ_excl_ = 0.04, 0.1, 0.2,0.4. (*e*) Plots of the mean standard deviation against γ_excl_. These standard deviation values measure the uncertainty of DDIP, as the input vectors to the DIPs are randomly initialized for each run. Larger γ_excl_ results in larger uncertainty.

We have demonstrated the crosstalk separation capability of our modified DDIP model in two cases, one with slice images reconstructed without using XRF data, the other reconstructed using the aid of XRF data but without fine tuning of phase retrieval parameters. In practice, one problem of concern might be the reproducibility of the algorithm due to its inherent uncertainty, which is contributed by the randomness of input vectors *z*_1_, *z*_2_, *z*_3_ and the random initialization of network parameters. In our experience, this uncertainty is associated with the value of γ_excl_, so we conducted a test to evaluate the change of result distribution with γ_excl_. On the Au/Ni dataset, we ran a series of DDIP separations using γ_excl_ = 0.04, 0.1, 0.2, 0.4, each run for 20 times. For the results of each γ_excl_, the standard deviation over the 20 runs at each pixel position is shown in Figs. 6 ▸(*a*)–6(*d*). The averages of these standard deviation maps are plotted in Fig. 6 ▸(*e*), which clearly show an increasing trend. Referring back to Fig. 3 ▸, the optimal result using shallow DIPs is obtained with γ_excl_ = 0.2, where the image mean is 0.68, but the uncertainty standard deviation is only around 0.04. In practice, one can also perform multiple runs and use the average *y*_1_ and *y*_2_ as the final results, so as to further decrease the uncertainty. Other than the detailed variation of the separated images, it is also possible for DDIP to undergo ‘slice confusion’: since the inputs to the generating DIPs are purely random, they do not inform DDIP that *y*_1_ should correspond to real features on slice 1, and vice versa for *y*_2_. If DDIP is confused about the slice arrangement, it may tend to generate the real, solid Au particles, which should lie on slice 1, on *y*_2_ instead. According to equation (3), these Au particles will be filtered by *f*_1_ to form *I*_1_, which is unphysical; the same would apply to *I*_2_. However, with a reasonable γ_excl_, this is very unlikely to happen: following the example above, if the real Au particles appear on *y*_2_, then they will appear unfiltered on *I*_2_; yet, on 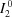 these particles are highpass filtered, and this leads to high mismatch loss which is unfavored. Therefore, the band loss of feature blending and our unsymmetrical use of *f*_1/2_ in the forward model drive the DDIP towards the correct slice arrangement. In our uncertainty test, we did not see slice confusion in all of our 80 separation results.

Both results shown in Section 3 involve two slices. In practice, multislice ptychography may be used to reconstruct three slices or more, and mutual crosstalk may involve more than two slices. In that case, one may add more DIPs, so that the number of image-generating DIPs matches the number of mutually crosstalking slices *N*. Meanwhile, the input and output channels of the weight-generating DIP may be increased to *N*, and the forward model of equation (3) may be expanded to *N* equations, constituting an *N* × *N* mixing matrix. Using too many DIPs will unavoidably impair the efficiency of the algorithm. However, in X-ray microscopy, the number of slices is typically small due to the large DoF of X-rays. Making the DDIP method more efficient for many-slice problems is a future direction to explore.

## 5. Conclusion   

Using a modified double-DIP architecture, we demonstrated the use of deep neural networks in mitigating the crosstalk artifacts of multislice ptychography phase retrieval. When the slice spacing is large (many multiples of the DoF), phase retrieval from scratch can provide slice reconstructions that are distinct from each other but affected by crosstalk, while post-processing using DDIP may suppress or remove the crosstalk on each slice. Combining multislice phase retrieval and DDIP can yield good reconstructions without XRF data in this case. When the slice spacing is small, phase retrieval may need the aid of XRF data in order to generate distinguishable slice images, and the retrieved images may still contain crosstalk artifacts without dedicated parameter tuning. One can also use DDIP in this case to suppress the crosstalk, so that one no longer has to spend time searching for the best values of phase retrieval hyperparameters. In order to account for the band loss of crosstalking features in a slice image, we pass them through a filtering function in our forward model. The filtering function can take the form of either a single convolutional filter or a shallow DIP. While the former is faster, the latter can often provide results with better preserved details. We expect that the findings will help improve the adaptability of multislice ptychography in imaging thick samples beyond the DoF limit.
